# Analysis of codon usage patterns in citrus based on coding sequence data

**DOI:** 10.1186/s12864-020-6641-x

**Published:** 2020-12-16

**Authors:** Zenan Shen, Zhimeng Gan, Fa Zhang, Xinyao Yi, Jinzhi Zhang, Xiaohua Wan

**Affiliations:** 1 High Performance Computer Research Center, Institute of Computing Technology, Chinese Academy of Sciences, Beijing, 100190 China; 2 University of Chinese Academy of Sciences, Beijing, 100000 China; 3 Key Laboratory of Horticultural Plant Biology (Ministry of Education), College of Horticulture and Forestry Science, Huazhong Agricultural University, Wuhan, 430070 China; 4 Department of Computer Science and Engineering, University of South Carolina, Colombia, 29201 USA

**Keywords:** Citrus, Codon usage, GC biology, Evolution, Correlation

## Abstract

**Background:**

Codon usage is an important determinant of gene expression levels that can help us understand codon biology, evolution and mRNA translation of species. The majority of previous codon usage studies have focused on single species analysis, although few studies have focused on the species within the same genus. In this study, we proposed a multispecies codon usage analysis workflow to reveal the genetic features and correlation in citrus.

**Results:**

Our codon usage analysis workflow was based on the GC content, GC plot, and relative synonymous codon usage value of each codon in 8 citrus species. This approach allows for the comparison of codon usage bias of different citrus species. Next, we performed cluster analysis and obtained an overview of the relationship in citrus. However, traditional methods cannot conduct quantitative analysis of the correlation. To further estimate the correlation among the citrus species, we used the frequency profile to construct feature vectors of each species. The Pearson correlation coefficient was used to quantitatively analyze the distance among the citrus species. This result was consistent with the cluster analysis.

**Conclusions:**

Our findings showed that the citrus species are conserved at the genetic level and demonstrated the existing genetic evolutionary relationship in citrus. This work provides new insights into codon biology and the evolution of citrus and other plant species.

## Background

The genetic code is degenerate. There are 64 different codons, including 61 codons encoding for amino acids and 3 stop codons, but only 20 translated amino acids. As a result of the degeneracy of the genetic code, many amino acids are encoded by two-to-six synonymous codons, termed condon usage bias. The genetic codes of different organisms are often biased towards the use of one of several codons. The codons that encode the same amino acid over the others are called synonymous codons [1]. These differences among the usage of the synonymous codons have been the important factor for the evolution of proteome diversity, and preferences for synonymous codons exists widely within the genomes due to mutation, natural selection, and random drift [2–4]. Thus, a comprehensive understanding of the biases in codon usage can help us explore the evolution of those proteins that have structural differences conserved at the sequence level [5–8].

Recently, studies based on full length ORF(open reading frame) sequences or genomes have shown wide variations in codon usage in many organisms. Most of these studies focused on single species such as *Escherichia coli* [9], *Caenorhabditis, Drosophila, Arabidopsis* [10], *Paeoniaceae lactiflora* [11] and *Megalobrama amblycephala* [12]. However, few studies has been performed on the correlation within the same genus based on codon usage patterns, and a similar study in citrus species was not based on the whole genome [13]. Therefore, further research and analysis of the Citrus genus could be useful for understanding the conservatism and evolution of different citrus species.

Citrus species are economically important evergreen trees that are major fruit producers in the world, with annual global yields of more than 130 million tons [14]. They are native to the subtropical and tropical regions of Asia and the Malay [15–17]. Citrus plants spread to Australasia, Japan and other regions during the early Pleistocene. The geographical origin, timing and dispersal of citrus species across southeast Asia remain unclear [18]. The investigation of genetic difference can help us get new insights on evolutionary relationship of citrus. To reveal the correlation in citrus species, we proposed a multispecies codon usage analysis workflow including data preprocessing, codon usage bias analysis, high-frequency codons identification of 8 different citrus species in this study. The difference between the same high-frequency codons among different citrus was no more than 0.05, and in 13 high-frequency codons, 11 of them were the same. Compared with other species in the plant kingdom, citrus showed similar codon usage bias. Moreover, pearson correlation coefficient was used to study the relationship among citrus quantitatively [19]. This can confirm the results of cluster analysis. The results will help us understand codon biology and evolution in citrus plants, and will help improve the research on correlation analysis of the same genus.

## Results and discussion

### Codon usage in 8 citrus genomes

The GC content may reflect significant compositional features of the genome. As the research shows, GC content still remained significantly negatively correlated with mean annual temperature, warmest and positively correlated with latitude and annual temperature range [20]. The average overall GC content in this study was 43.67%, and varied among the different citrus species and codon positions. *Citrus grandis* showed the highest GC content with a value of 43.79%, *Citrus sinensis* showed the lowest GC content with a value of 43.50%. For GC content at the first position, which obtained the highest value in citrus, *Atlantia buxifolia* showed the highest value at 50.70% and *Citrus reticulata ‘Mangshan’* showed the lowest value at 50.51%. The highest and lowest values of GC2, GC3 and GC3s were GC2: 40.56%(*Citrus grandis*) and 40.12%(*Citrus sinensis*); GC3: 40.28%(*Citrus clementina*) and 39.35%*(Atlantia buxifolia)*; and GC3s: 38.02%*(Citrus clementina)* and 37.08%(*Citrus reticulata ‘Mangshan’*). Among the 8 citrus species, the value of GC3 and GC3s of *Atlantia buxifolia* was the lowest (*Atlantia buxifolia* is known as Chinese box orange and was formerly named *Severinia buxifolia*) [21]. The GC base pair is more thermally stable than AT base pair, and it can reflect the distribution history in citrus species. As an example of a primitive citrus species, *Atlantia buxifolia* showed that codon usage was not completely conserved and evolution was more active (Table 1).

**Table 1:** GC content of CDS across 8 Citrus Species

**Citrus Species**	**Variety**	**Genes**	**GC%**	**GC**_**1**_**%**	**GC**_**2**_**%**	**GC**_**3**_**%**	**GC**_**3**__***s***_**%**	**ENC**
*Atlantia buxifolia*	Atalantia	59755	43.51	50.70	40.47	39.35	37.08	52.48
*Fortunella hindsii*	Mandarin	48789	43.80	50.85	40.53	40.01	37.74	52.47
*Citrus grandis*	Pummelo	38039	43.79	50.67	40.56	40.13	37.87	52.55
*Citrus sinensis*	Sweet	40773	43.50	50.52	40.12	39.87	37.59	52.44
*Citrus medica*	Citron	40808	43.70	50.63	40.49	39.98	37.70	52.63
*Citrus reticulata ‘Mangshan’*	Mandarin	36852	43.59	50.51	40.32	39.94	37.66	52.65
*Citrus ichangensis*	Papeda	36936	43.77	50.58	40.54	40.20	37.93	52.59
*Citrus clementina*	Mandarin	29687	43.73	50.57	40.35	40.28	38.02	52.83
Average	-	41455	43.67	50.63	40.42	39.97	37.70	52.58

Genes represents the number of sequences after filtering; GC1, GC2 and GC3 represent the GC content of the first, second, third base of codon; GC3s represents the GC content of the third synonymous position; ENC represents the effective number of codons

### Neutrality plot analysis

**Fig. 1:**
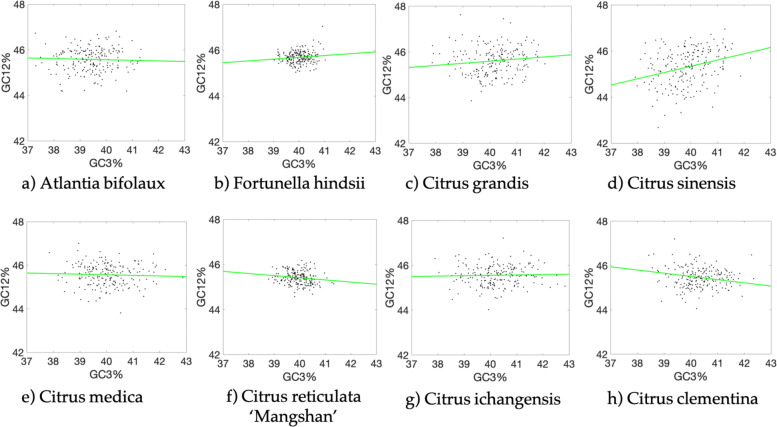
Neutrality plot of 8 citrus species. The green solid line represents the regression line. **a** Atlantia buxifolia, the regression line is *y*=−0.0258*x*+46.5950,*R*^2^=0.0418. **b** Fortunella hindsii, the regression line is *y*=0.0781*x*+42.5627,*R*^2^=0.1218. **c** Citrus grandis, the regression line is *y*=0.0921+41.9104,*R*^2^=0.1288. **d** Citrus sinensis, the regression line is *y*=0.2712*x*+34.4916,*R*^2^=0.3047. **e** Citrus medica, the regression line is *y*=−0.0275*x*+46.6589,*R*^2^=0.0494. **f** Citrus reticulata ‘Mangshan’, the regression line is *y*=−0.0954*x*+49.2216,*R*^2^=0.1476. **g** Citrus ichangensis, the regression line is *y*=0.0174*x*+44.8579,*R*^2^=0.0341. **h** Citrus clementina, the regression line is *y*=−0.2456*x*+51.3338,*R*^2^=0.0341

The neutrality plot was used to analyze the relationships among the three codon positions to examine the role of mutation in citrus [22]. We found that citrus genes had a narrow range of GC12(42%~48%) and GC3(36%~42%) values and there were significiant correlations between GC12 and GC3 in *Citrus sinensis* and *Citrus clementina*, where the slope of the regression line was more than 0.2. The significantly correlation indicating that the GC mutation bias effect the GC contents similarly among all positions of codons. In contrast, there was no significantly correlations in other 6 citrus species, and the slope of regression line was near 0, indicating there are low mutation bias or high conservation of GC content and limited evidence of directional mutation pressure in these citrus genes. The results also showed that *Citrus sinensis* was the most affected species by directional mutation pressure due to its highest correlation coefficient of 0.3047 in citrus (Fig. 1). Because of the partially silent nature of the third codon position, GC3 represents one of the most neutral nucleotides within the genome with respect to the G + C content [23].

### ENc plot analysis

**Fig. 2:**
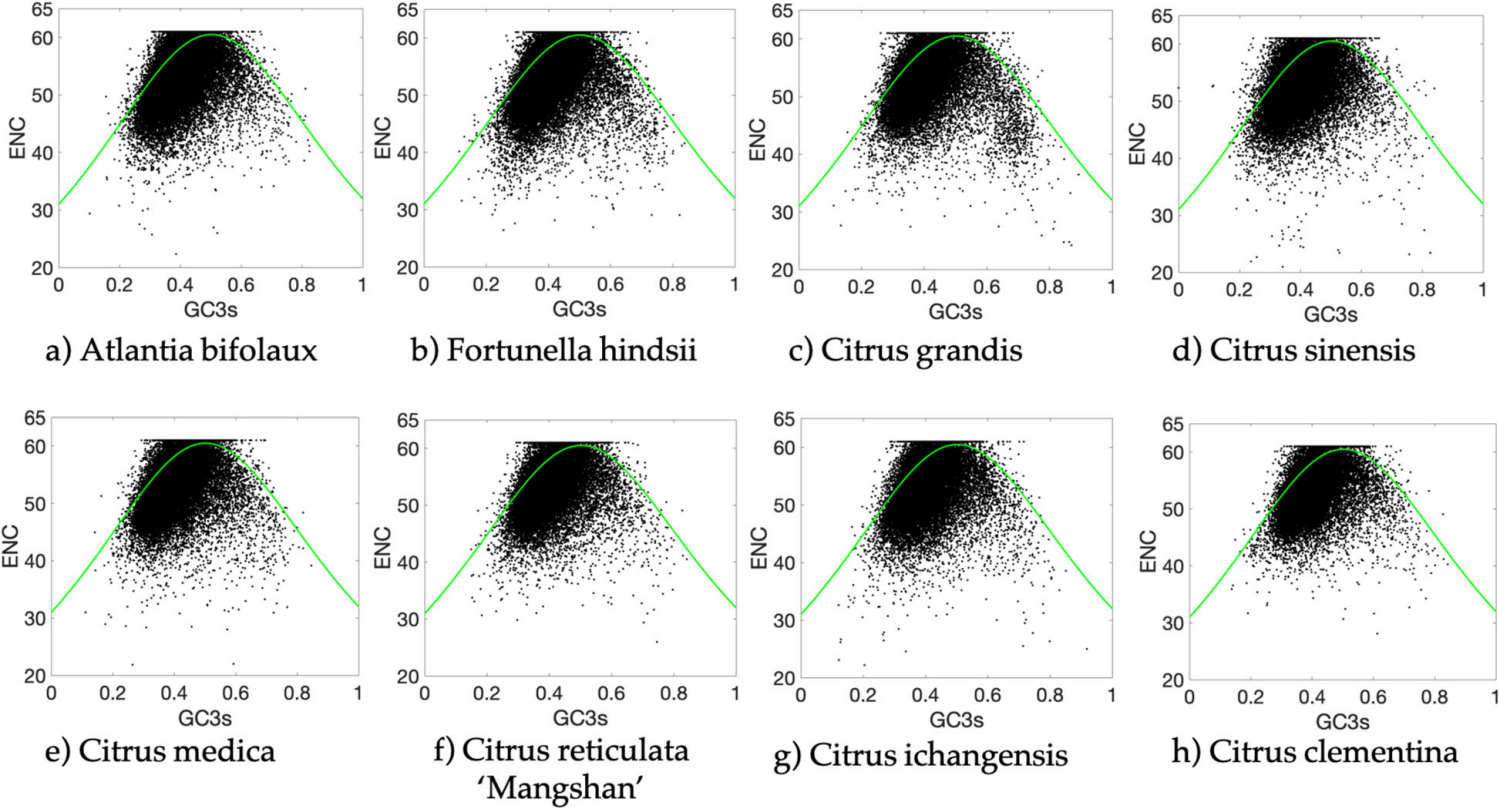
Neutrality plot of 8 citrus species. ENCs were plotted against GC content at the third position. The green solid line represents the expected curve of positions of genes when the codon usage was only determined by the GC3s composition. **a** Atlantia buxifolia. **b** Fortunella hindsii. **c** Citrus grandis. **d** Citrus sinensis. **e** Citrus medica. **f** Citrus reticulata ‘Mangshan’. **g** Citrus ichangensis. **h** Citrus clementina

Analysis of the relation between GC3 and ENC can determine the relation between the differences in ENC and the differences in GC contents. The ENc-plot is an effective tool to study the codon usage patterns, and it was used here to explore the influence of GC3s on the codon bias in citrus [24]. As shown in Fig. 2, citrus species showed similar patterns in ENc plot. Most genes were located below the expected ENc-plot curve, whereas only a small number of genes were at or above the curve. These results indicated that the conditional mutation might be a weak factor in shaping the codon bias, which is also affected by other factors.

**Fig. 3:**
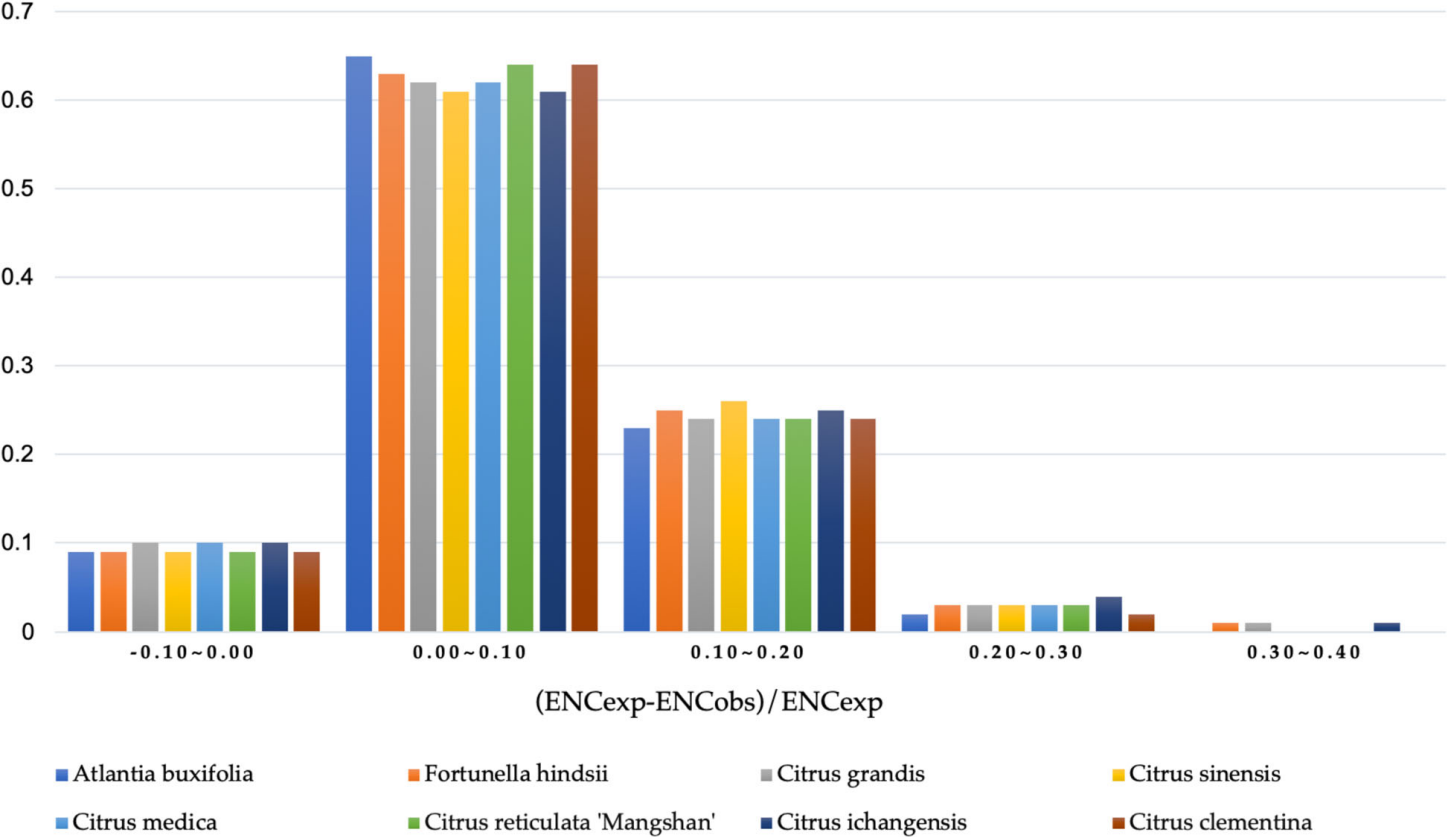
Frequency distribution of (ENCexp-ENCobs)/ENCexp. ENCexp represents expected ENC values and ENCobs represents ENC observed values. The peak located in 0 to 0.1

To further prove the conservative of the influence of GC3s in citrus and to validate the difference between the observed and expected ENC values, (ENCexp-ENCobs)/ENCexp was calculated. As shown in Fig. 3, there was a single peak, the shape and location of the peak were similar among the citrus species. More than 60% of the total genes of the 8 citrus species were distributed within the 0 to 0.1 range of the (ENCexp-ENCobs)/ENCexp values, indicating that the most actual ENC values were slightly smaller than the expected ENC values from the GC3s. These results also prove that the conditional mutation might be a weak factor affecting the evolution history of citrus.

### High-Frequency codons and codon pairs usage analysis in citrus

The RSCU of codons was calculated. AGA was the most frequent codon, which encoded Arg. GCT and GTT were the next two highly frequent codons, which encoded Ala and Val, respectively. Of all the 8 citrus species, AGA, GTT, GCT, TCT, TTG, ATT, GAT, CAT, AAT, TTT and TAT were identified as the most frequent codons in common. Among these codons, 91% ended with A/T, and only 9% of them ended with G/C, indicating that citrus species were more likely to use A/T at the third position of high-frequency codons. Among the high-frequency codons, 36.4% started with G/C and the other 63.6% started with A/T, indicating a bias towards A/T at the first position of the high-frequency codons. *Atlantia buxifolia* had the most high-frequency codons at 15. It is possible that the GC to AT mutation in *Atalantia buxifolia* mainly occurred during the evolution (Table 2) [25].

**Table 2:** The top five high-frequency codons

Citrus Species	codon(RSCU)					N
*Atlantia buxifolia*	AGA(1.93)	GCT(1.70)	GTT(1.68)	TCT(1.61)	TTG(1.55)	15
*Fortunella hindsii*	AGA(1.89)	GCT(1.62)	GTT(1.63)	TCT(1.56)	TTG(1.54)	11
*Citrus grandis*	AGA(1.93)	GCT(1.65)	GTT(1.65)	TCT(1.56)	TTG(1.54)	11
*Citrus sinensis*	AGA(1.96)	GCT(1.66)	GTT(1.65)	TCT(1.57)	TTG(1.54)	12
*Citrus medica*	AGA(1.95)	GCT(1.66)	GTT(1.66)	TCT(1.58)	TTG(1.54)	14
*C.reticulata ‘Mangshan’*	AGA(1.97)	GCT(1.66)	GTT(1.66)	TCT(1.57)	TTG(1.55)	13
*Citrus ichangensis*	AGA(1.95)	GCT(1.66)	GTT(1.65)	TCT(1.57)	TTG(1.54)	13
*Citrus clementina*	AGA(1.94)	GCT(1.66)	GTT(1.65)	TCT(1.57)	TTG(1.55)	13

N: the number of high-frequency codons of each citrus species

The RSCU of four NCG codons in the citrus species were the lowest (CCG:0.46 TCG:0.43 ACG:0.42 GCG:0.32). The results showed that citrus have a relatively high methylation level. Four NTA codons also had a low RSCU value (TTA:0.84 ATA:0.77 GTA:0.65 CTA:0.56), as low RSCU values of NTA codons inhibit mRNA degradation and thus increases protein production [26].

In practice, codon pairs are used more frequently. At the mRNA translation level, codon pair context influences the speed and accuracy of translation processes, and are species specific. Single codon optimization does not mean global optimization. Codon pairs also show some bias among synonymous pairs. As shown in the Additional file 1, based on 3,721 (61*61) codon pairs, 832 high-frequency codon pairs were identified on average, and *Atlantia buxifolia* had the highest number of high-frequency codon pairs at 839, and *Citrus grandis* had the lowest number of pairs at 822. The last three codon pairs were nnGCnn, nnCCnn and nnCTnn, which may relate to a lower methylation level of citrus DNA [27]. This result was consistent with our hypothesis that the codon usage patterns in *Atlantia buxifolia* was not completely conserved in the evolutionary process.

### Codon usage patterns across the plant kingdom

**Fig. 4:**
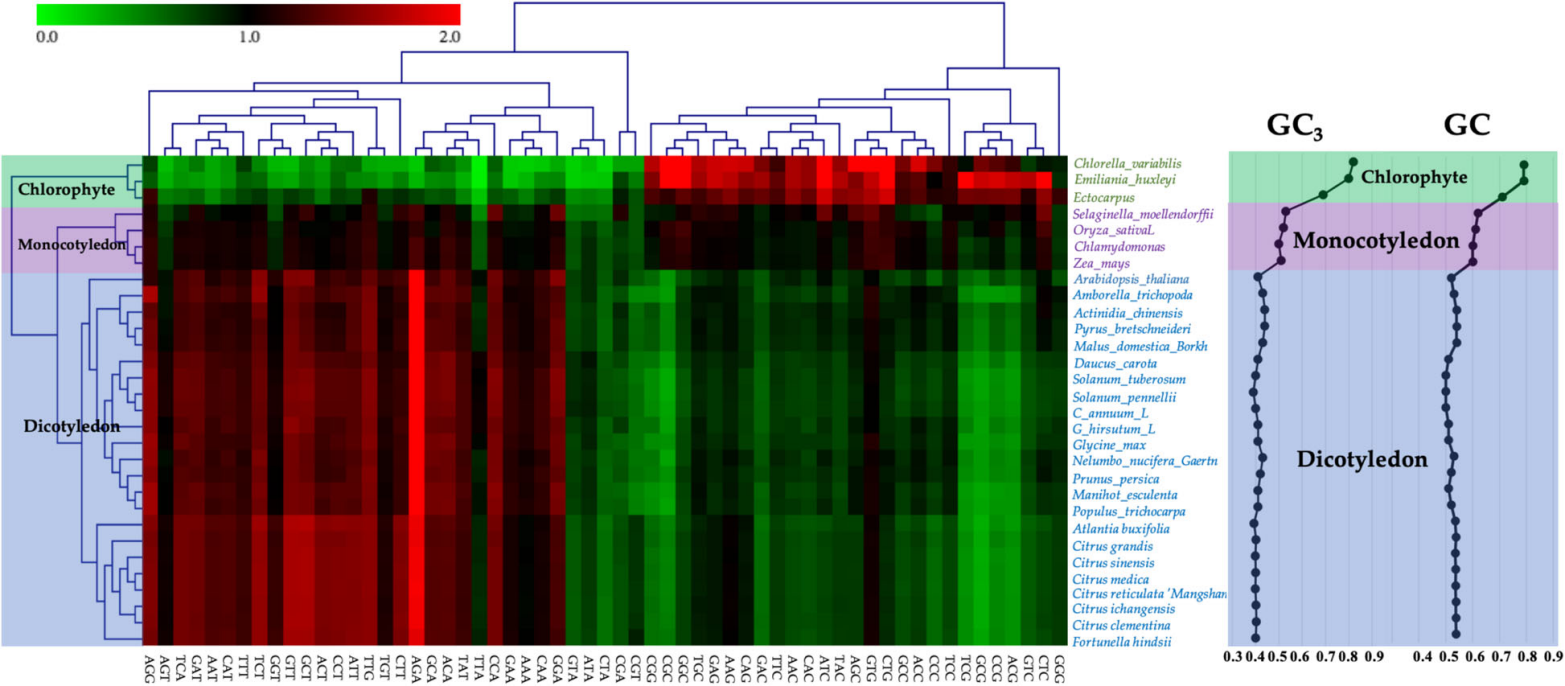
Heat map of RSCU of 59 codons from 30 species using Euclidean distance and average clustering module. GC and GC3 distribution in ORFs from 30 plant genomes

The natural selection distinguishing between synonymous codons constrains the rate of nucleotide substitution. And within an evolutionary framework, the degree of codon bias reflects a balance between selection and synonymous mutations [28]. A heat map via biclustering was used to describe the variations of codon usage bias among 8 citrus species and 22 other plant species based on the RSCU of all 59 synonymous codons. The clustering results indicated that all of the 30 plants could be divided into three groups. The original Chlorophyte plants were clustered together. Monocotyledon plants were grouped together and included *Selaginella moellendorffii*, *Oryza sativaL*, *Brachypodium distachyon*, *Chlamydomonas* and *Zea mays*. Dicotyledon plants were clustered into the third group and included citrus species, *Camellia sinensis* and *Opulus trichocarpa* [29]. Citrus species had a closer relationship than other dicotyledon species (Fig. 4).

To prove the species in the same group had the similar GC and GC3 contents, GC distribution from 30 plant genomes was plotted. And they varied greatly in different species and have changed during evolution, which was confirmed by the results (Fig. 4). The original single-celled or multi-celled Chlorophyte plants had very high GC3 contents (0.69 to 0.82), whereas in the monocotyledons, the GC3 content decreased but was still over 0.5, and in Dicotyledons, the GC3 content was approximately 0.4. It is hypothesized that one of the major selective advantages of GC-rich DNA is the ability for more complex gene regulation [20].

### Pearson correlation coefficient among citrus species

**Fig. 5:**
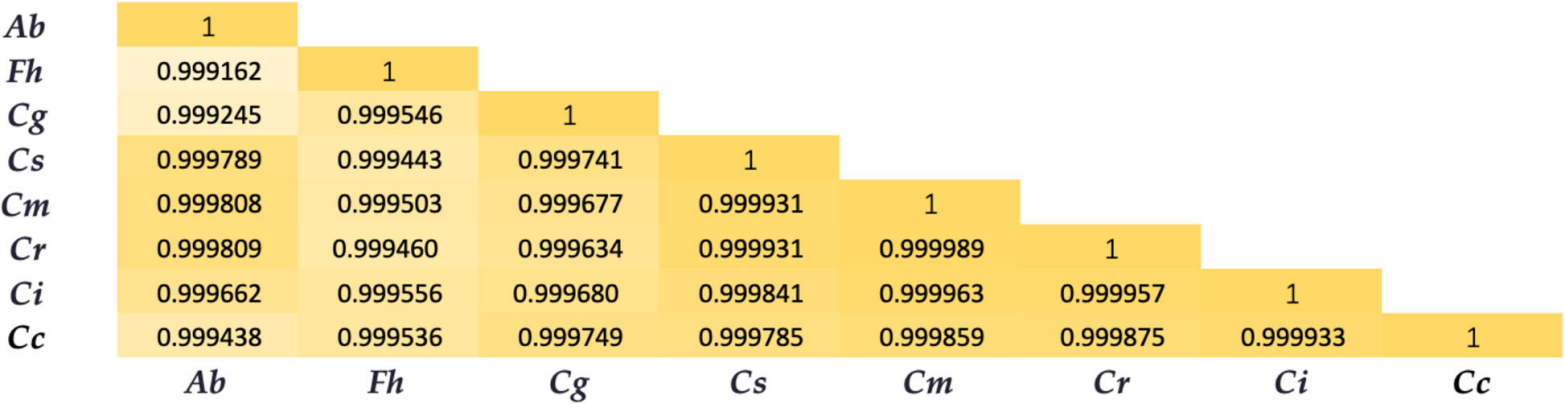
Heat map of pearson correlation coefficient among Citrus species. Ab: Atlantia buxifolia; Fh: Fortunella hindsii; Cg: Citrus grandis; Cs: Citrus sinensis; Cm: Citrus medica; Cr: Citrus reticulata ‘Mangshan’; Ci: Citrus ichangensis; Cc: Citrus Clementina

The similarity among citrus species was calculated quantitatively based on Pearson correlation coefficients, which were used to construct heat maps. The heat map of Pearson correlation coefficients between each species is shown in Fig. 5, which illustrates the correlation among citrus and shows which pairs of species have close relationships.

*Citrus medica* and *Citrus reticulata ‘Mangshan’* had the highest value of 0.999989. This result was confirmed by the cluster analysis, which showed that these two species were clustered together. *Citrus medica* and *Citrus ichangensis* also clustered together, with a Pearson value of 0.999957. *Atlantia buxifolia* and *Fortunella hindsii* had the lowest value at 0.999162 and were the last pair clustered together. This result can also be confirmed biologically, as *Citrus reticulata* and *Citrus medica* are both ancestral species. The wild *Mangshan ’mandarin’* and *Citrus reticulata* are the parents of *Citrus reticulata ‘Mangshan’* [30], providing a closer relationship compared to other citrus species.

## Conclusion

We identified a multispecies codon usage analysis workflow that revealed the genetic features and correlation of the genus Citrus. In particular, we performed a comprehensive analysis of codons and codon pair usage in 8 citrus species and 22 other plants. Our results showed few differences in codon features among citrus species and, thus, that the genomes of citrus species were conserved. Regarding GC content, the nucleotide content of citrus genes was slightly GC poor and AT rich. As for Pearson correlation coefficient of dinucleotide sequence profile among citrus species, its results can also be confirmed by the cluster analysis. Using this workflow, we compared 8 species of citrus. This method can also be used on other species. However, our results should be considered cautiously, as more data are required. Future work will focus on additional codon usage indices in citrus to determine if citrus is conserved at these levels.

In conclusion, our findings provided insight into the codon usage patterns of citrus species and could be used for the cloning and expression of exogenous genes in citrus and other functionally important plants.

## Methods

### Sequence data collection and filtering

The dataset consisted of two main parts. Firstly, the protein-coding sequences(*.cds.fa.gz) of 8 citrus species were downloaded from the CAP (Citrus sinensis Annotation Project) database (http://citrus.hzau.edu.cn/orange/index.php). Secondly, the compared genome and annotation data (*_genomic.fna.gz, *_genomic.gff.gz) of 22 published plant species including 15 dicot species, 4 monocot species and 3 chlorophyte species were downloaded from NCBI Genome database (https://www.ncbi.nlm.nih.gov/genome).

Protein-coding sequences (CDS) of those compared plant species were extracted by Tbtools(http://cj-chen.github.io/tbtools/). All CDS without an AUG start codon, not ending with UAA, UAG or UGA stop codons, and having uncertain nucleotides and containing internal stop codons were filtered out, which were regarded as low quality sequences because of invalid format. After filtering, the remaining high quality sequences were used for further analysis. The filtering procedure was performed by python scripts written in-house.

### Indices of codon usage

The overall GC content and the GC content at the first, second and third position reflect the strength of directional mutation. RSCU is an index used to study the overall synonymous codon usage variation among genes. Codons with RSCU values over 1.0 were identified at a high frequency and codons with RSCU values below 1.0 showed negative codon usage bias. RSCU was calculated according to the formula described in Sharp and Li [31]. The ENC reflects the degree of codon bias for 20 amino acids across ORFs. The ENC was between 20 and 61. An ENC value close to 20 indicates that only one of the synonymous is preferred, and a value close to 61 shows that each synonymous codon is used equally. The GC content and RSCU were calculated with C++ programs written in-house, and the ENC was calculated using the codonW1.4.4 (http://codonw.sourceforge.net/).

### Overview of the codon usage analysis workflow

**Fig. 6:**
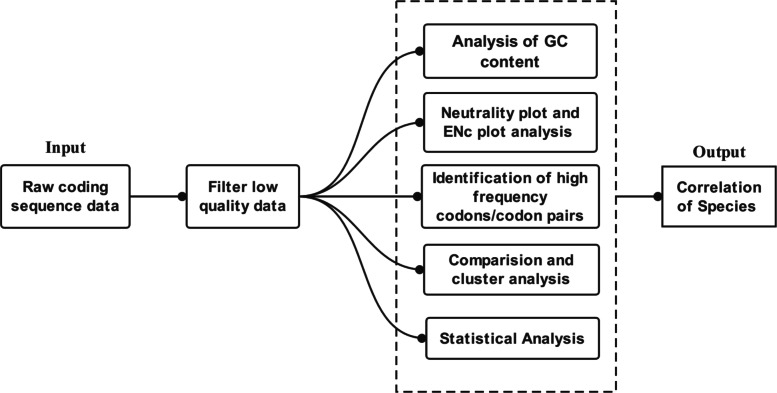
Process of workflow

Our workflow consists of six parts: data preprocessing, GC content analysis, neutrality plot and ENc plot analysis, high-frequency codons identification, comparison and cluster analysis, and statistical analysis. We examined the correlation of citrus species based on codon usage patterns (Fig. 6).

#### Analysis of gC content

GC content includes the overall GC content, GC1 (GC content of 1st nucleotide in codon), GC2 (GC content of 2nd nucleotide in codon), GC3 (GC content of 3rd nucleotide in codon) and GC3s (GC content of 3rd synonymous codons). The GC content reveals GC bias and varies greatly between species [32]. An analysis of codon usage pattern can provide a basis for understanding the relevant mechanism of the biased usage of synonymous codons. This analysis also has both practical and theoretical applications for understanding the basics of molecular biology [33].

#### Neutrality plot and eNc plot analysis

A neutrality plot (GC12-GC3) was used to estimate and characterize the codon usage patterns among three codon positions. GC12 represents the average of GC1 and GC2. A plot regression with a slope of 0 indicates no effect of directional mutation pressure (complete selective constraints), whereas a slope of 1 indicates the same mutation module between GC12 and GC3 and that complete neutrality was the main factor in evolution [11].

The ENc-plot(ENC-GC3s) is a general strategy to determine whether the codon usage of a gene is affected by mutation and selection. The expected ENc values were plotted against the GC3s values and were calculated according to Equation 1, where F represents the frequency of the estimated GC3s. That the actual ENC values lie on or around the standard GC3s curve indicates that the codon bias is determined by a G + C mutation bias only. In other words, the values distributed far below the standard curve shows that other factors such as selection effects are present [34].

1 $$ ENc = 2 + F + \frac{29}{F^{2} + (1-F)^{2}}  $$

#### Identification of high-Frequency codons and codon pairs

Those codons with RSCU values over 1.5 or having a relative frequency above 60% of the synonymous codons for the corresponding amino acids were identified as high-frequency codons. Codon pairs with the last codon coding the same amino acid were defined as synonymous codon pairs. High-frequency codon pairs were defined as those codons with RSCPU (relative synonymous codon pair usage) values over 1.5 or when the number of codon pairs included over 60% of the total number of synonymous codon pairs [35–37]. The novel equation to compute RSCPU for a pair of codon is as follows:

2 $$ RSCPU_{i} = \frac{x_{i}}{\frac{1}{n_{i}} \sum_{i=1}^{n_{i}}{x_{i}}}  $$

where *x*_*i*_ is the number of the occurrences of the *i*^*th*^ kind of codon pairs, and *n*_*i*_ is the number of synonymous codon pair for the *i*^*th*^ type amino acid pair[38]. Identification of high-frequency codons and codon pairs were performed by C++ programs written in-house.

#### Comparison and cluster analysis

The RSCU of 59 codons (excluding the 3 stop codons and codons with synonymous codons) of 8 citrus species and 22 other plants were clustered using the Mev4.8.1 software (https://sourceforge.net/projects/mev-tm4/) [39]. The hierarchical clustering, Euclidean distance and sample tree parameters were set to cluster with the RSCU. The GC and GC3 variation of 30 different species were analyzed using Microsoft Excel.

#### Statistical analysis

The distribution characteristics of dinucleotides can be used to study nucleic acids [40]. To further estimate the correlation among citrus species, we extracted the dinucleotide frequency profile vectors. Four kinds of nucleotides make up 16 different dinucleotide feature vectors. Each feature vector was calculated according to equation *f*_*xy*_=*MN*/(*L*−1), where *f*_*xy*_ stands for the frequency of each nucleotide pair, M and N stand for the kinds of nucleotides, *MN* stands for the number of occurrences of the dinucleotides and L represents the length of all sequences.

For each sequence, we used a two bit sliding window to obtain the frequency of the vectors. Thus, each nucleic acid was calculated twice, and equation *p*_*xy*_=*f*_*xy*_/(*f*_*x*_*f*_*y*_) was used to avoid repeated calculations based on the above-mentioned results. Variable *p*_*xy*_ represents the frequency profile of the dinucleotides. Variable *p*_*x*_ and *p*_*y*_ represent the corresponding frequency profile of the nucleic acids [41].

The 16 different kinds of dinucleotides represent the signature of the species. We used the Pearson correlation coefficient to calculate the distance and obtain the similarity between two species. The Pearson correlation coefficient *r* was defined as follows:

3 $$ r=\frac{\sum{XY}-\frac{\sum{X}\sum{Y}}{N}}{\sqrt{\left(\sum{X^{2}}-\frac{(\sum{X})^{2}}{N}\right) \left(\sum{Y^{2}}-\frac{(\sum{Y})^{2}}{N}\right)}}  $$

where *X* and *Y* represent the set of each dinucleotides frequency vectors of the citrus species. N represents the number of the points. Here, N equals to 16.

## Supplementary information

**Additional file 1** High frequency codon pairs table. The RSCPU value and number of each high frequency codon pairs of each citrus species.

## Data Availability

The datasets of 8 citrus species analysed are available in the CAP (Citrus sinensis Annotation Project) database (http://citrus.hzau.edu.cn/orange/download/index.php). The datasets of other 22 plants analysed are available in the NCBI Genome database (ID: 694, 2, 79022, 411, 10, 16337, 12, 4, 12031, 16401, 12793, 358, 860, 400, 24150, 10896, 10704, 5, 14095, 388, 441, 98) (https://www.ncbi.nlm.nih.gov/genome/).

## Abbreviations

Ab: Atlantia buxifolia
Cc: Citrus Clementina
CDS: Coding sequence
Ci: Citrus ichangensis
Cg: Citrus grandis
Cm: Citrus medica
Cr: Citrus reticulata ‘Mangshan’
Cs: Citrus sinensis
ENC: Effective number of codons
Fh: Fortunella hindsii
RSCU: Relative synonymous codon usage
RSCPU: Relative synonymous codon pair usage
